# Initiation and Completion of Adjuvant Chemotherapy After Total Versus Partial Pancreaticoduodenectomy for Pancreatic Cancer

**DOI:** 10.1245/s10434-025-18378-3

**Published:** 2025-09-23

**Authors:** Romy Weber, Filippa Lara Maria Kuehni, Pauline Aeschbacher, Stéphanie Fabienne Perrodin, Andreas Andreou, Hanna Kaibel, Nina Marie Rohrmeier, Martin D. Berger, Beat Gloor, Anna Silvia Wenning

**Affiliations:** 1 Department of Visceral Surgery and Medicine, Inselspital, Bern University Hospital, University of Bern, Bern, Switzerland; 2 Department of General, Visceral and Transplant Surgery, University Hospital Münster, Münster, Germany; 3 Department of Medical Oncology, Inselspital, Bern University Hospital, University of Bern, Bern, Switzerland

## Abstract

**Background:**

Partial pancreaticoduodenectomy (PD) followed by adjuvant chemotherapy (AC) is standard treatment for resectable pancreatic ductal adenocarcinoma (PDAC) of the pancreatic head. Total pancreatectomy (TP) has historically been reserved for extensive tumors or salvage procedures due to concerns about morbidity and quality of life (QoL). However, recent evidence shows comparable perioperative outcomes and QoL between TP and PD. The authors hypothesized that avoiding postoperative pancreatic fistula TP would achieve AC initiation and completion rates similar to those for PD, even in more complex patients.

**Methods:**

This study retrospectively analysed all patients who underwent TP or PD for PDAC at the authors’ center between 2014 and 2021. Rates, timing, and completion of AC were compared. The decision for TP versus PD was based on patient and intraoperative factors at the discretion of the surgeon.

**Results:**

Of 263 included patients, 74 underwent TP and 189 underwent PD. Total pancreatectomy was performed mainly for repetitive positive resection margins or splenic vessel involvement (59 %). The TP patients had more comorbidities (liver disease, 16.2 % vs 5.8 % *p* = 0.013; diabetes, 40.5 % vs 24.9 % *p* = 0.016), longer surgeries (7.2 vs 6 h; *p* = 0.001), more vascular reconstructions (77 % vs 50.8 %; *p* = 0.001), and greater blood loss (1200 vs 600 ml; *p* = 0.001). Despite these factors, morbidity and mortality were comparable. The two groups did not differ in rates of AC initiation (66 % vs 76 %; *p* = 0.156), completion (69.4 % vs 74.1 %; *p* = 0.578), and timing (median, 7 weeks in both groups; *p* = 0.533).

**Conclusion:**

Despite higher surgical complexity, AC initiation and completion rates after TP were comparable with those after PD. With modern diabetes management, TP represents a valid surgical option for selected high-risk patients without compromising oncologic treatment.

Pancreatic ductal adenocarcinoma (PDAC) is increasing in incidence and is expected to become the second leading cause of cancer-related deaths within a decade.^1^ Tumor resection without multimodal treatment has yielded poor outcomes, with a disease-free survival (DFS) of 6 months and a 5-year survival rate of 10 %.^2^ In contrast, resection combined with adjuvant FOLFIRINOX chemotherapy has improved median DFS to 21.4 months and 5-year survival to 43.2 %.^3^ In addition, for resectable PDAC, a recent randomized trial analyzing neoadjuvant chemotherapy followed by resection showed no survival benefit.^4^ Consequently, adjuvant chemotherapy (AC) currently is the standard care after PDAC resection, and the percentage of patients receiving AC is an important parameter of treatment quality and adherence to current guidelines.

Adjuvant chemotherapy rates after PDAC resection vary. Prospective clinical trials of well-selected patients report chemotherapy completion rates between 60 %^2^ and 89 %,^3,5^ whereas real-world data indicate initiation of AC for only 62 %, with a median start 8.5 weeks after surgery and completion rates as low as 18.6 % in some studies.^6,7^ Key risk factors for the omission or delay of adjuvant therapy include surgical complications,^7–12^ which are associated with poorer oncologic outcomes. Neoadjuvant chemotherapy, intended to mitigate these issues, has shown no oncologic benefit in resectable pancreatic head cancer.^4^ Therefore, surgical resection with minimal morbidity is crucial for timely initiation of AC.

The most significant complication after partial pancreaticoduodenectomy (PD) is a postoperative pancreatic fistula (POPF) due to insufficient anastomotic healing.^13^ According to the definition of the International Study Group, POPF is classified as biochemical leak (BL) grade B or C.^14^ Although various techniques and interventions to prevent POPFs (e.g., different anastomotic methods and drainage systems) have been studied, no single approach has consistently proved to be superior.^15,16^

Total pancreatectomy (TP) offers a way to eliminate the risk of POPF. Historically, primary TP was a procedure reserved for large tumors or resections requiring complex vascular reconstructions.^17^ Outcomes for these patient populations were generally poor, and managing diabetes type 3c was challenging. However, advancements such as continuous glucose monitoring and automated insulin delivery have simplified diabetes management and improved QoL for TP patients. Whereas an analysis of the nationwide German diagnosis-related group statistics showed a mortality rate of 15.6 % for 7056 patients after standard TP,^18^ other recent studies have shown no significant differences in mortality, morbidity, or short-term QoL between TP and PD.^19–22^ These findings have contributed to a reduced threshold among pancreatic surgeons for performing primary TP in recent years. Despite strong evidence supporting comparable short-term outcomes, data on AC after TP remain limited.

This study aimed to characterize patients undergoing TP and PD and to investigate the initiation, timing, and completion of AC after TP versus PD for PDAC.

## Methods

### Study Design and Population

A retrospective study analyzed all patients undergoing PD or TP with curative intent for PDAC at the University Hospital of Bern, Switzerland between 2014 and 2021. Data collection was approved by the local ethics community (ID 2023-0566) for all patients who signed the general consent form.

All the patients underwent a standardized preoperative evaluation comprising medical history, blood tests, and radiologic imaging, including computed tomography (CT) scan and magnetic resonance imaging (MRI). All the patients were discussed in a multidisciplinary tumor meeting, and surgical resection was recommended after assessment of local tumor resectability in the absence of metastases. Patients with locally advanced tumor stages, defined as arterial infiltration or encasement greater than 180° and venous infiltration, which does not allow venous reconstruction, were scheduled for neoadjuvant chemotherapy before resection.

### Endpoints

The primary endpoint was the rate of AC initiation. The secondary endpoints were rate of AC completion, timing to AC initiation, cycles of administered chemotherapy, postoperative morbidity and mortality, and overall survival (OS).

### Surgical Procedures

Patients who had undergone oncologic PD and TP with radical lymphadenectomy were included in the analysis. The laparoscopic technique for PD and TP was introduced in 2017 in our center. Since then, the decision for open or laparoscopic access has been made by the main surgeon pre- or intraoperatively. Robotic pancreatic head resections were introduced after 2021 and thus were not included in this analysis.

The following decision factors for TP were applied pre- or intraoperatively: comorbidities, tumor location including splenic vessel invasion, positive resection margins at the level of the pancreatic neck, no relevant pancreatic remnant for anastomosis, high-risk anastomosis in combination with vessel reconstruction or signs of insufficient resolution after endoscopic retrograde cholangiopancreatography (ERCP), pancreatitis in the pancreatic remnant, or relevant mucinous cystic lesions in the pancreatic remnant. Vascular resection and reconstruction (direct anastomosis or graft interposition) were performed for vascular invasion. Drains were placed to monitor POPF or postoperative bleeding. During TP, the spleen was preserved whenever possible (no tumor involvement or injury of the splenic vein or artery).

After surgery every patient was admitted to an intermediate care unit (IMCU) for one night to receive monitoring and postoperative care. A POPF was detected by elevated lipase levels in perianastomotic drains and classified according to the definition of the International Study Group.^14^ The drains were removed when fluid quality was serous and the lipase levels in the fluid were low. Patients requiring a completion pancreatectomy in the postoperative course remained in the PD group for analysis.

### Chemotherapy

Recommendations for neoadjuvant or adjuvant chemotherapy were determined by the multidisciplinary tumor board. In general, neo-adjuvant chemotherapy was reserved for borderline or locally advanced tumors.^23^ The administration of both neoadjuvant and adjuvant chemotherapy was documented, including the start of therapy within weeks after surgery, type of medication, modifications, number of cycles, and completion status. Additionally, we investigated the reasons why some patients did not receive adjuvant chemotherapy.

### Study Variables

Preoperative patient characteristics including gender, age, comorbidities, body mass index (BMI), history of smoking or alcohol consumption, and American Society of Anesthesiology (ASA) score were recorded. We documented the perioperative characteristics with regard to the operation (surgical technique, duration of surgery, intraoperative blood loss, and vascular reconstruction), and with regard to the postoperative course (length of intermediate care unit [IMCU] and hospital stays, readmission, morbidity, mortality, and reoperation including salvage pancreatectomy). The time of the IMCU stay was defined as time from the day of surgery to the day of transfer to the surgical ward. The hospital length of stay was documented in days. Readmission within 90 days after surgery was defined as a new hospital admission after discharge.

Postoperative morbidity included any complication after surgery within 90 days. We documented surgical complications such as POPF, re-operation, postoperative hemorrhage, and surgical-site infections as well as pulmonary, cardiovascular, renal, and thromboembolic complications. Major complications were defined using the Clavien-Dindo classification as grade IIIa or higher.^24^ Postoperative mortality was defined as any death within the first 30 days after surgery.

Histopathologic specimens were classified according to the tumor-node-metastasis (TNM) classification of malignant tumors.^25^ In addition, tumor grading and resection status were assessed. Resection status was investigated by microscopic analysis of the resection margins (bile duct, retropancreatic tissue, vascular groove or resected vein, and pancreatic neck [in PD]). An R0 resection was defined if surgical margins were ≥1 mm microscopically negative for malignant cells.

### Statistical Analysis

Continuous variables are presented as medians and ranges, whereas categorical variables are presented as absolute numbers and percentages. Comparisons between the groups were performed using Fisher's exact test or the chi-square test for categorical variables and Mann-Whitney *U* test or Student’s *t* test for continuous variables, as appropriate.

We planned the following subgroup analyses: (1) early/late chemotherapy start, defined as application of the first cycle of adjuvant chemotherapy within 8 weeks (early) after surgery or later (late), and (2) chemotherapy regimen, starting in 2018, when another adjuvant chemotherapy strategy, mFOLFIRINOX, was demonstrated to lead to prolonged survival compared with gemcitabine and implemented in oncologic routine for patients younger than 75 years with an Eastern Cooperative Oncology Group (ECOG) score of 0 or 1.^26^ Kaplan-Meier curves and log-rank were used to assess OS and DFS.

Statistical analyses were performed using EZR (version 1.61; Jichi Medical University Saitama Medical Center) and R software (version 4.3.1; R Foundation for Statistical Computing).

## Results

**Fig. 1:**
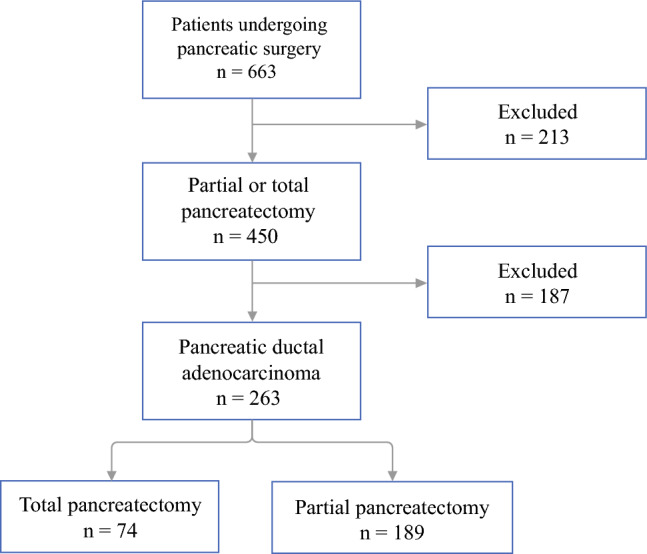
Flow chart of the patient selection

Of 663 patients undergoing pancreatic surgery at our institution between 2014 and 2021, 263 met the inclusion criteria. Of these patients, 74 (28 %) underwent TP and 189 (72 %) underwent PD (Fig. 1).

### Patient Characteristics (Table 1)

The TP group had more liver diseases (16.2 % vs 5.8 %; *p* = 0.013) and insulin-dependent diabetes (40.5 % vs 24.9 %; *p* = 0.016) than the PD group. The TP group also showed a trend toward a higher BMI (26 vs 24 kg/m^2^; *p* = 0.053).

**Table 1:** Patients’ characteristics

Variable	TP (*n* = 74) *n* (%)	PD (*n* = 189) *n* (%)	*p* value
*Gender*			
Female	33 (44.6)	89 (47.1)	0.784
Male	41 (55.4)	100 (52.9)	
Median age: years (range)	72 (36–87)	69 (37–84)	0.227
Age >70 years	42 (56.8)	87 (46.0)	0.132
Cardiovascular disease	42 (56.8)	104 (55.0)	0.890
Kidney disease	15 (20.3)	36 (19.0)	0.863
Pulmonary disease	17 (23.0)	39 (20.6)	0.738
Infectious disease	4 (5.4)	3 (1.6)	0.100
Liver disease	12 (16.2)	11 (5.8)	**0.013**
Neurologic disease	10 (13.5)	17 (9.0)	0.269
Diabetes	30 (40.5)	47 (24.9)	**0.016**
Median BMI: kg/m^2^ (range)	26.00 (16–43)	24.00 (15–46)	0.053
BMI >30 kg/m^2^	11 (14.9)	23 (12.2)	0.546
Smoking	23 (31.1)	55 (29.1)	0.765
Alcohol consumption	16 (21.6)	41 (21.7)	1.000
ASA status			0.359
ASA 2	13 (17.6)	39 (20.6)	
ASA 3	51 (68.9)	135 (71.4)	
ASA 4	10 (13.5)	15 (7.9)	

Bold values indicate statistical significance (*p* < 0.05)

*TP* total pancreatectomy, *PD* partial pancreaticoduodenectomy, *BMI* body mass index

### Perioperative Variables (Table 2)

The TP group had fewer minimally invasive resections (6.8 % vs 27.5 %; *p* < 0.001), longer operations (7.2 vs 6.0 h; *p* < 0.001), higher rates of vascular reconstructions (77.0 % vs 50.8 %; *p* < 0.001), and greater blood loss (1200 vs 600 ml; *p* < 0.001).

**Table 2:** Perioperative variables

Variable	TP (*n* = 74) *n* (%)	PD (*n* = 189) *n* (%)	*p* value
Laparoscopic-assisted technique	5 (6.8)	52 (27.5)	**<0.001**
Median operating time: hours (range)	7.20 (4.0–11.45)	6.00 (3.7–9.45)	**<0.001**
Vascular reconstruction	57 (77.0)	96 (50.8)	**<0.001**
Venous reconstruction	53 (71.6)	86 (45.5)	**<0.001**
Arterial reconstruction	18 (24.3)	17 (9.0)	**0.002**
Median blood loss: ml (range)	1200 (200–8600)	600 (100–3500)	**<0.001**
Median IMCU stay: days (range)	2 (0–11)	1 (0–37)	**<0.001**
Median hospital stay: days (range)	16 (2–70)	15 (3–61)	0.159
Readmission within 90 days	11 (15.3)	21 (11.2)	0.401
In-hospital mortality within 30 days	4 (5.4)	5 (2.6)	0.274
Postoperative morbidity	45 (60.8)	122 (64.6)	0.572
Major postoperative morbidity	24 (32.4)	49 (25.9)	0.289
Postoperative hemorrhage	7 (9.5)	23 (12.2)	0.668
Superficial/deep surgical site infection (%)	16 (21.6)	53 (28.0)	0.350
Organ/space surgical-site infection	9 (12.2)	12 (6.3)	0.132
Pulmonary complication	12 (16.2)	18 (9.5)	0.135
Cardiovascular complication	12 (16.2)	12 (6.3)	**0.017**
Renal complication	8 (10.8)	2 (1.1)	**0.001**
Thromboembolic complication	9 (12.2)	12 (6.3)	0.132
Re-operation	11 (14.9)	31 (16.4)	0.853
POPF			
None		157 (83.1)	
Biochemical leak		13 (6.9)	
Type B		6 (3.2)	
Type C		13 (6.9)	
Completion pancreatectomy		14 (7.4)	

Bold values indicate statistical significance (*p* < 0.05)

*TP* total pancreatectomy, *PD* partial pancreaticoduodenectomy, *IMCU* intermediate care unit, *POPF* postoperative pancreatic fistula

The IMCU stay was longer in the TP group (2 days vs 1 day; *p* < 0.001). Overall postoperative morbidity did not differ significantly between the groups. However, the TP group experienced significantly more cardiovascular (16.2 % vs 6.3 %; *p* = 0.017) and renal (10.8 % vs 1.1 %; *p* = 0.001) complications.

After PD, 17 % (*n* = 32) of the patients experienced a POPF, including 10.1 % (*n* = 19) clinically significant POPFs (type B, 3.2 %; type C, 6.9 %) leading to completion pancreatectomy for 7.4 % (*n* = 14) of the patients.

### Tumor Characteristics (Table 3)

The TP patients showed a trend toward a higher neoadjuvant chemotherapy rate (13.5 % vs 6.3 %; *p* = 0.081; Table 4). The study found no significant difference in the rates of AC initiation (67 % vs 77 %; *p* = 0.156), its completion (69 % vs 74 %; *p* = 0.578), or the number of administered cycles (6 vs 6; *p* = 0.088).

**Table 3:** Tumor characteristics

Variable	TP (*n* = 74) *n* (%)	PD (*n* = 189) *n* (%)	*p* Value
*TNM status*			
T1	0 (0.0)	12 (6.3)	**0.022**
T2	31 (41.9)	87 (46.0)	0.583
T3	43 (58.1)	89 (47.1)	0.131
T4	0 (0.0)	1 (0.5)	1.000
*Nodal status*			
N0	13 (17.6)	40 (21.2)	0.609
N1	35 (47.3)	105 (55.6)	0.272
N2	26 (35.1)	44 (23.3)	0.062
*Grade*			
Well-differentiated	7 (9.5)	19 (10.1)	0.977
Moderately differentiated	36 (48.6)	93 (49.2)	
Poorly differentiated	28 (37.8)	71 (37.6)	
Not applicable	3 (4.1)	6 (3.2)	
*Resection status*			
R0	45 (62)	133 (72)	0.135
R1	27 (37.0)	51 (27.6)	0.175
R2	1 (1.4)	1 (0.5)	0.487

Bold values indicate statistical significance (*p* < 0.05)

*TP* total pancreatectomy, *PD* partial pancreaticoduodenectomy, *TNM* tumor-node-metastasis

The TP group had no T1 stage disease (0 % vs 6.3 %; *p* = 0.022). The TP group showed a trend toward a higher N2 status (35.1 % vs 23.3 %; *p* = 0.062), although this difference did not reach statistical significance.

### Neoadjuvant and Adjuvant Chemotherapy (Tables 4 and 5)

**Table 4:** Chemotherapy data

Variable	TP (*n* = 74) *n* (%)	PD (*n* = 189) *n* (%)	*p* Value
Neoadjuvant chemotherapy	10 (13.5)	12 (6.3)	0.081
No AC due to completed neoadjuvant chemotherapy	1 (1.3)	1 (0.5)	n/a
Adjuvant chemotherapy	49 (67)	144 (76.6)	0.156
Chemotherapy completion if started	34 (69.4)	109 (74.1)	0.578
No. of chemotherapy cycles (all chemotherapy strategies) (range)	6 (1–12)	6 (1–17)	0.088
Weeks from surgery to chemotherapy (range)	7 (3–12)	7 (3–16)	0.533
Adjuvant mFOLFIRINOX initiated	14 / 19 (74)	41 / 54 (76)	0.847
Adjuvant mFOLFIRINOX completed	8 /14 (57)	30 / 41 (73)	0.525
Reasons for no adjuvant chemotherapy:	17 (68)	23 (51.1)	0.446
Medical reasons	6 (24)	16 (35.6)	
Patient refused	1 (4)	5 (11.1)	
Unknown			

*TP* total pancreatectomy, *PD* partial pancreaticoduodenectomy, *AC* adjuvant chemotherapy, *n/a* not applicable

**Table 5:** Early versus late start of AC for 45 patients in the TP group and 135 patients in the PD group whose exact date of treatment start was documented^a^

Early vs late start	TP *n* (%)	PD *n* (%)	Total *n* (%)
Late start (>8 weeks)	12 (26.67)	25 (18.52)	37 (20.56)
Early start (≤8 weeks)	33 (73.33)	110 (81.48)	143 (79.44)
Total	45 (100.00)	135 (100.00)	180 (100.00)

*TP* total pancreatectomy, *PD* partial pancreaticoduodenectomy

^a^Pearson chi-square test: χ^2^ (1) = 1.3721; *p* = 0.241

The first cycle of chemotherapy was applied an average of 7 weeks after the pancreatic resection in both groups. When mFOLFIRINOX was introduced as efficient adjuvant treatment for PDAC in patients younger than 76 years in 2018, all the patients were evaluated for this regimen by the treating oncologist except for one patient in each group. In the TP group, 14 of the 19 patients (74 %) who qualified for mFOLFIRINOX subsequently received this type of AC. In the PD group, the corresponding figures were 41 (76 %) of 54 patients.

Three patients in the TP group and five patients in the PD group did not qualify for mFOLFIRINOX due to a reduced postoperative ECOG score. Other reasons for not choosing mFOLFIRINOX were comorbidities and age. The reasons for not receiving AC were similarly distributed in the two groups. The leading cause was that patients were not fit for chemotherapy or not willing to undergo additional chemotherapeutic treatment. One patient in each group had received the full neoadjuvant chemotherapy and therefore did not qualify for AC.

**Fig. 2:**
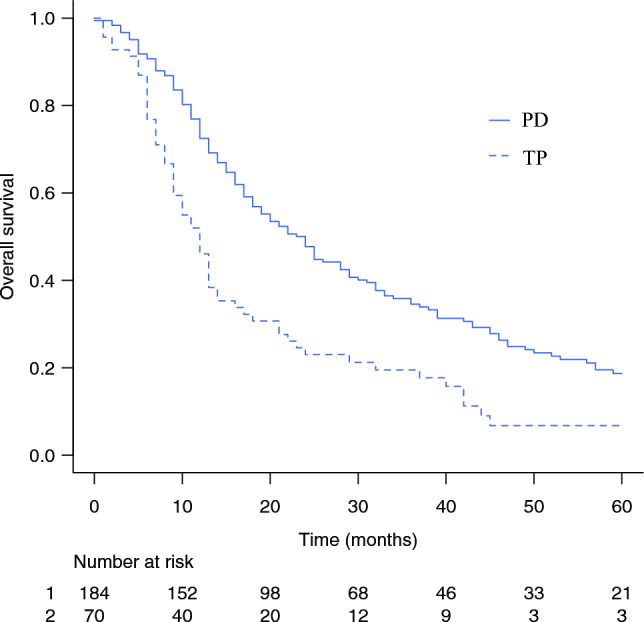
Kaplan-Meier curve for overall survival of patients after total pancreatectomy (TP) and partial pancreaticoduodenectomy (PD)

**Fig. 3:**
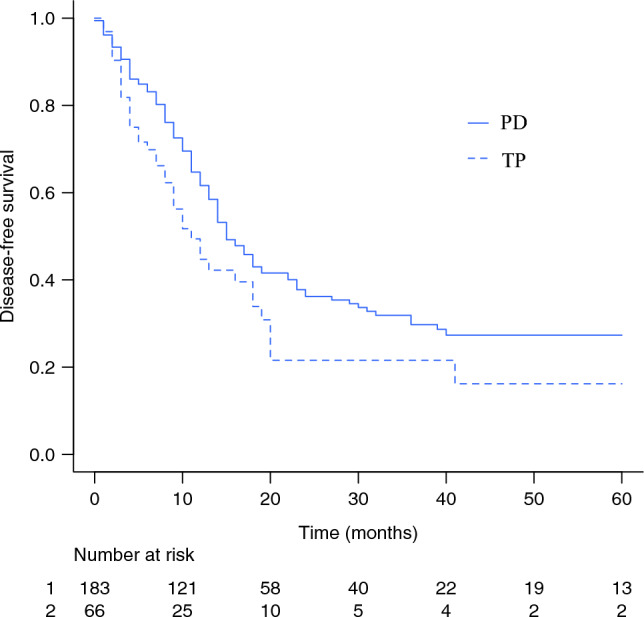
Kaplan-Meier curve for disease-free survival of patients after total pancreatectomy (TP) and partial pancreaticoduodenectomy (PD)

For those patients with a precisely documented start of AC, we compared early (<8 weeks after surgery) and late (≥8 weeks after surgery) initiation of AC and found no significant difference (*p* = 0.241) between the TP and PD groups (Table 5). The TP group had shorter OS (12 vs 24 months; *p* < 0.001) and DFS (11 vs 15 months; *p* = 0.041) (Figs. 2 and 3).

## Discussion

This study compared outcomes of patients with PDAC undergoing TP and PD, focusing particularly on the administration of AC in routine clinical practice. We observed similar AC initiation rates in the two groups (67 % after TP vs 77 % after PD; *p* = 0.156). Although lower than the guidelines recommendations advocating AC for all resected PDAC patients,^27^ our findings are consistent with real-world reports. For example, a retrospective study from Germany documented AC rates of 44 % after TP and 53 % after PD,^28^ whereas a Dutch study reported an AC rate of 62 % after pancreatic resections, including distal pancreatectomy.^8^

Recovery after surgery plays a pivotal role in determining AC initiation. In our cohort, comparable rates of major morbidity and mortality likely explain the similar AC rates. Previous studies demonstrated that postoperative complications reduce the likelihood of a patient receiving AC and are associated with worse survival,^8^ but this effect alone does not fully account for the poorer outcomes we observed in the TP group.

Adjuvant chemotherapy completion rates, number of administered cycles, and receipt of mFOLFIRINOX also were comparable between the groups, suggesting that despite higher surgical complexity, TP patients were not disadvantaged in accessing systemic therapy. This contrasts with earlier concerns that TP patients, due to more challenging recovery and diabetes management, might be less likely to receive or tolerate chemotherapy.^29^ The high proportion of TP patients who received mFOLFIRINOX further underscores improved perioperative care and postoperative fitness, which is encouraging given the significant survival benefits of this regimen.^26^

Despite these similarities in AC administration, OS and DFS survivals were shorter for the TP patients. A recent German cancer registry analysis with 756 patients per group similarly found poorer long-term survival after TP than after PD.^28^ In our cohort, the TP patients had more advanced disease, longer operations, greater blood loss, and higher rates of vascular reconstruction. These factors likely reflect more aggressive tumor biology and a heavier disease burden, which AC cannot fully offset. This interpretation is supported by evidence that re-resection to achieve negative margins, a common indication for TP, does not consistently improve outcomes, likely due to the underlying biologic aggressiveness of the disease.^30^

Our R0 resection rate was comparable with registry data,^28^ yet survival remained inferior for the TP patients. In addition, the TP patients experienced more cardiovascular and renal complications. These findings may be attributable to higher baseline comorbidity as well as to the more extensive surgical procedures required, especially greater blood loss and the resulting fluid and hemodynamic imbalances. Although TP eliminates the risk of POPF, the trade-off may be greater systemic morbidity that influences long-term outcomes.

It is important to interpret our findings within the limitations of this retrospective, single-center study. Patient selection bias was possible, particularly because TP was predominantly chosen in cases of advanced local tumor involvement. The relatively smaller TP group also may have limited the ability to detect subtle differences. Nevertheless, our results provide valuable insights into the real-world feasibility of TP.

In summary, TP has historically been regarded as more radical and morbid than PD, but our findings, together with recent evidence, suggest that it is a feasible option for carefully selected patients. Comparable AC initiation and completion rates indicate that TP does not hinder the delivery of systemic therapy. Advances in diabetes management, including continuous glucose monitoring and automated insulin delivery, have further improved long-term QoL after TP.^31^ Taken together, these findings support TP as a valid surgical strategy for high-risk or complex PDAC cases, in which achieving a safe and complete resection without compromising access to adjuvant therapy is paramount. Prospective multicenter studies are warranted to further define survival and QoL outcomes after TP versus PD.
